# Predominant cleavage of proteins N-terminal to serines and threonines using scandium(III) triflate

**DOI:** 10.1007/s00775-019-01733-7

**Published:** 2019-10-30

**Authors:** Christian J. Koehler, Bernd Thiede

**Affiliations:** Department of Biosciences, University of Oslo, P.O. Box 1066, Blindern, 0316 Oslo, Norway

**Keywords:** Endoproteinase, Lewis acid, Mass spectrometry, Protein cleavage

## Abstract

**Abstract:**

Proteolytic digestion prior to LC–MS analysis is a key step for the identification of proteins. Digestion of proteins is typically performed with trypsin, but certain proteins or important protein sequence regions might be missed using this endoproteinase. Only few alternative endoproteinases are available and chemical cleavage of proteins is rarely used. Recently, it has been reported that some metal complexes can act as artificial proteases. In particular, the Lewis acid scandium(III) triflate has been shown to catalyze the cleavage of peptide bonds to serine and threonine residues. Therefore, we investigated if this compound can also be used for the cleavage of proteins. For this purpose, several single proteins, the 20S immune-proteasome (17 proteins), and the Universal Proteomics Standard UPS1 (48 proteins) were analyzed by MALDI–MS and/or LC–MS. A high cleavage specificity N-terminal to serine and threonine residues was observed, but also additional peptides with deviating cleavage specificity were found. Scandium(III) triflate can be a useful tool in protein analysis as no other reagent has been reported yet which showed cleavage specificity within proteins to serines and threonines.

**Graphic abstract:**

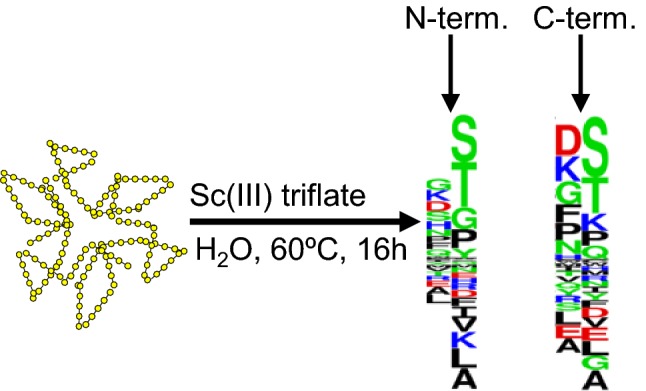

**Electronic supplementary material:**

The online version of this article (10.1007/s00775-019-01733-7) contains supplementary material, which is available to authorized users.

## Introduction

Site-selective cleavage of proteins is an important tool for the analysis of protein sequences. Trypsin cleaves proteins C-terminal to arginine and lysine residues, and is by far the most commonly used endoproteinase in bottom-up proteomics [1]. However, protein sequences can contain either large sequence regions without arginines and lysines or where these amino acids are too close together. In such cases, peptides suitable for analysis by mass spectrometry cannot be obtained using trypsin. Other endoproteinases cleave either at basic (Arg-C, Lys-C, Lys-N, and LysargNase), acidic (Asp-N and Glu-C), or hydrophobic (chymotrypsin) amino acids [2–4]. Only few chemical reagents for the selective cleavage of proteins have been reported [5]. The most often used chemical reagent, cyanogen bromide, selectively cleaves peptides bonds on the C-terminal side of methionines, but the reagent is volatile and toxic and thus only occasionally used [6]. Metal-catalyzed specific hydrolysis of peptide bonds has been described, but their practical use in protein sequencing is still in infancy [7]. The site-selective cleavage of peptides and proteins primarily at X–Y bonds in X–Y-His and X–Y-Met sequences by Pd(II) complexes and Met-Z bonds by Pt(II) complexes was reported [8, 9]. Asparagine-selective bond cleavage of N-terminal protected peptides using diacetoxyiodobenzene under mild conditions has been reported as well [10]. Selective cleavage of peptide bonds N-terminal to serines has been described using *N*,*N*′-disuccinimidyl carbonate [11], and a water-soluble-organoradical conjugate [12]. Truncation of long-lived proteins at the N-terminal side of serines and threonines has been observed [13]. Zinc is a major trace metal in biological systems and it could be shown that the presence of Zn(II) catalyzes this truncation process [14, 15]. Different metal complexes were compared and revealed that Sc(III) triflate is particularly effective in cleaving peptide bonds at serine and threonine residues [16]. Peptides with up to 42 amino acids were investigated and revealed fragments due to cleavage at serine and threonine residues. In this report, we evaluated the use of Sc(III) triflate for the cleavage of proteins (10–80 kDa) using MALDI–MS and LC–MS. The obtained mass spectrometrical data allowed a more detailed analysis of the cleavage specificity. Predominant cleavage only N-terminal to serine and threonine residues was determined, but also many peptide fragments were identified due to semi- and non-specific cleavage.

## Materials and methods

### Cleavage of proteins using Sc(III) triflate

Sc(III) triflate was applied to the protein samples at different temperatures (30 °C, 40 °C, 50 °C, 60 °C, and 70 °C), reaction times (1 h, 4 h, 16 h, and 40 h), buffers (100 mM ammonium acetate, 100 mM ammonium bicarbonate, 100 mM ammonium citrate, 100 mM potassium dihydrogen phosphate, 100 mM sodium carbonate, 100 mM MES, 100 mM phosphate-buffered saline, 100 mM triethylammonium carbonate, 0.1% tifluoroacetic acid, and water), and Sc(III) triflate molarity (10 mM, 100 mM, and 1 M) Further experiments were performed with 100 mM Sc(III) triflate at 60 °C for 16 h in water. All buffers and proteins were purchased from Sigma-Aldrich (Oslo, Norway).

### MALDI–mass spectrometry

MALDI–MS was performed as previously described [17]. Briefly, a MALDI–TOF/TOF (Ultraflex II, Bruker Daltonics, Bremen, Germany) was used. The samples were analyzed in the TOF mode for the generation of peptide mass fingerprints. α-Cyano-4-hydroxycinnamic acid (20 mg/mL) in 0.3% aqueous trifluoroacetic acid/acetonitrile (2:1) was used as matrix.

### LC–MS

LC–MS was performed as previously described [18]. An LC/MS system consisting of a Dionex Ultimate 3000 RSLCnano-LC system (Sunnyvale CA, USA) connected to a linear quadrupole ion trap—Orbitrap (LTQ-Orbitrap XL) mass spectrometer (ThermoElectron, Bremen, Germany) equipped with a nanoelectrospray ion source was used to analyze the tryptic peptides. For liquid chromatography separation, an Acclaim PepMap 100 column (C18, 3 µm, 100 Å) (Dionex, Sunnyvale CA, USA) capillary of 25 cm bed length was used with a flow rate of 300 nL/min. Two solvents A (0.1% formic acid) and B (aqueous 90% acetonitrile in 0.1% formic acid) were used to eluate the peptides from the nano column. The gradient went from 3 to 35% B in 40 min and from 35 to 50% B in 3 min and finally to 80% B in 2 min. The mass spectrometer was operated in the data-dependent mode to automatically switch between Orbitrap-MS and LTQ–MS/MS acquisition. Survey full-scan MS spectra (from *m*/*z* 300 to 2000) were acquired in the Orbitrap with resolution *R* = 60,000 at *m/z* 400 and allowed the sequential isolation of the top six ions, depending on signal intensity, for fragmentation on the linear ion trap using collision-induced dissociation at a target value of 10,000 charges.

### Data analysis

LC–MS data were acquired using Xcalibur v2.5.5 and raw files were processed to generate peak list in Mascot generic format (*.mgf) using ProteoWizard release version 3.0.331. Database searches were performed using Mascot in-house version 2.4.0. A fragment ion mass tolerance of 0.5 Da, parent ion tolerance of 10 ppm, oxidation of methionines, and acetylation of the protein N-terminus as variable modifications were considered as search parameters for all analyses. For full- and semi-specific cleavage at serine and threonine residues, two missed cleavage sites were applied, whereas no missed cleavage site was used for the database search without enzymatic cleavage specificity. The mass spectrometry proteomics data have been deposited to the ProteomeXchange Consortium via the PRIDE [19] partner repository with the data set identifier PXD014918.

## Results and discussion

### Predominant cleavage of proteins N-terminal to serines and threonines using Sc(III) triflate

**Fig. 1:**
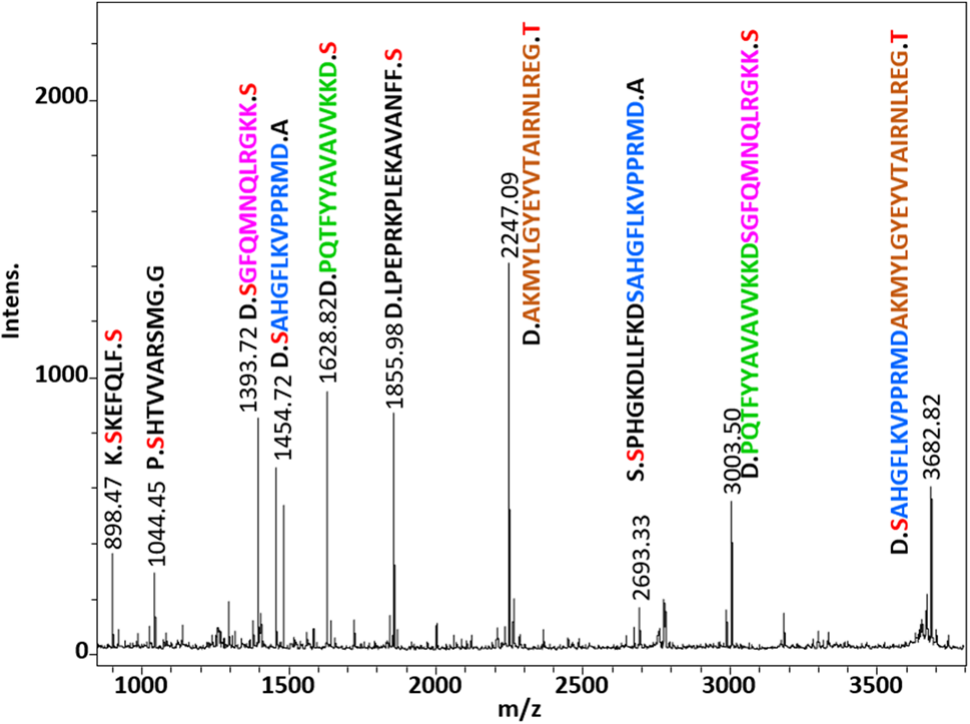
Peptide mass fingerprint of human transferrin using Sc(III) triflate. The analysis was performed with MALDI–TOF–MS (Ultraflex II, Bruker Daltonics) using 1 pmol of transferrin. The corresponding peptide sequences obtained by LC–MS are shown including previous and following amino acid. Serine and threonine residues due to N-terminal cleavage are displayed with red letters. The most intense peaks corresponded to peptides after full- or semi-specific N-terminal cleavage of serine and threonine residues

**Fig. 2:**
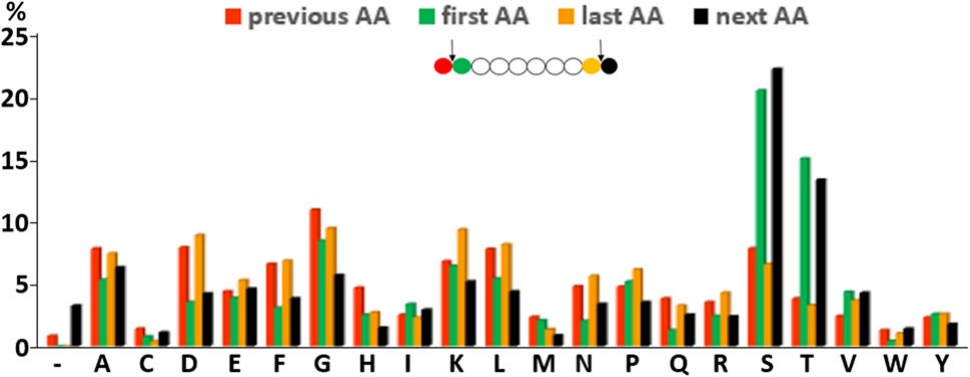
Sc(III) triflate cleavage specificity. 12 standard proteins revealed 2403 unique identified peptide sequences after LC–MS analysis. The percentages of the cleavage sites at the protein termini (-) and at all 20 amino acids (AAs) including previous and next amino acid are presented

**Fig. 3:**
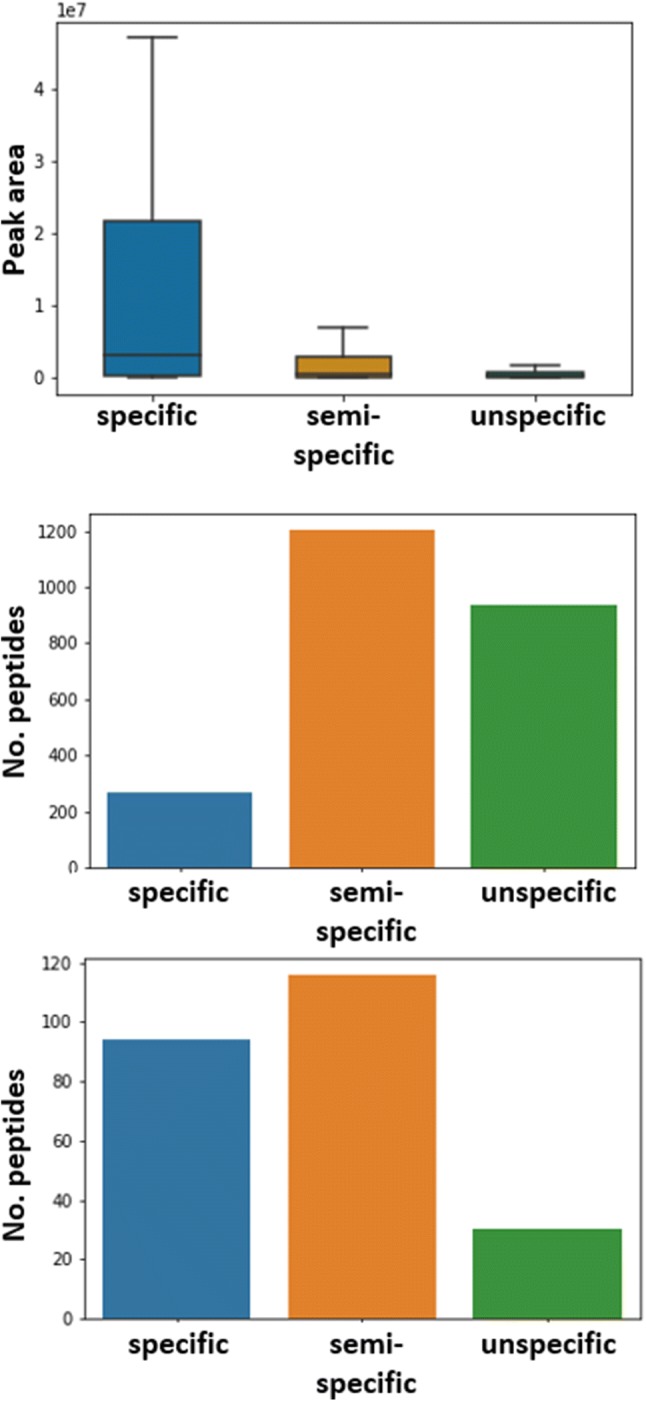
Degree of cleavage specificity of Sc(III) triflate. The box plot at the top shows the relation of the specificity of peptides concerning their intensity (intense outliers were excluded for visibility reasons). The bar plot in the middle displays the absolute distribution of all identified peptides (2403 peptides). In the box plot at the bottom, the 10% most intense peptides (240 peptides) were considered and the distribution is clearly biased towards peptides specifically cleaved N-terminal to serine and threonine residues

Different reaction conditions using Sc(III) triflate for the digestion of proteins were tested first with two proteins [alpha-1-glycoprotein (bovine), and beta-casein (bovine)]. Out of the different tested temperatures (30 °C, 40 °C, 50 °C, 60 °C, and 70 °C), reaction times (1 h, 4 h, 16 h, and 40 h), buffers (100 mM ammonium acetate, 100 mM ammonium bicarbonate, 100 mM ammonium citrate, 100 mM potassium dihydrogen phosphate, 100 mM sodium carbonate, 100 mM MES, 100 mM phosphate-buffered saline, 100 mM triethylammonium carbonate, 0.1% trifluoroacetic acid, and water), and Sc(III) triflate molarity (10 mM, 100 mM, 1 M), highest scores after database searches using Mascot were obtained for 60 °C/16 h/water/100 mM Sc(III) triflate. To further investigate if Sc(III) triflate can cleave proteins in general, these reaction conditions were applied to ten other proteins (alpha-casein (bovine), concanvalin (Jack bean), alpha-crystallin (bovine), cytochrome c (horse), glyceraldehyde-3-phosphate dehydrogenase (rabbit), beta-lactoglobulin (bovine), myoglobin (horse), ribonuclease A (bovine), thioredoxin (*E. coli*), and transferrin (human); all from Sigma-Aldrich). The reaction products of 1 pmol starting material were analyzed by MALDI–MS and LC–MS, respectively. The peptide mass fingerprints recorded by MALDI–MS revealed that the most intense peaks were almost exclusively cleavage products due to N-terminal cleavage at serine and threonine residues with full or semi-specificity (Fig. 1 and supplementary Fig. 1). Strikingly, several overlapping sequences were observed in most of the MALDI–MS spectra (Supplementary Fig. 1). A possible explanation for this observation could be that the proteins were first cleaved specifically and then further degraded. Searching the LC–MS data with three different cleavage specificities (no specificity, semi-specific, and full-specific cleavage at N-terminal serine and threonine residues) disclosed that the cleavage products were not exclusively products of N-terminal cleavage to serines and threonines. Actually, higher identification scores were achieved at semi and no enzyme specificity compared to full N-terminal cleavage at serines and threonines (Table 1). However, favored cleavage at these sites was observed considering the identified 2403 unique peptides as revealed by motif analysis using GibbsCluster (Supplementary Fig. 2) [20]. In addition, the percentages of the amino acids around the cleavage sites were calculated (Fig. 2). The proteins were cleaved in around 35% of identified peptides N-terminally to serine and threonine residues and none of the other amino acids showed preferred cleavage. As a note, cleavage C-terminal to aspartic acid was observed for Aβ1-42 as minor intense fragments, suggesting that a hard carboxylate functional group directs Sc(III) for cleavage of the peptide bonds. Our data set agrees with this observation, because C-terminal cleaved peptides at aspartic acids were observed twice as often as for N-terminal cleavages. As with MALDI–MS, the LC–MS signals detected with highest intensities belonged to peptides cleaved N-terminal to serine and threonine residues (Fig. 3).

**Table 1:** Single proteins were incubated with 100 mM Sc(III) triflate

Protein	Score/SC full (cut-off 5)	Score/SC semi (cut-off 14)	Score/SC no (cut-off 20)
α1-Acid glycoprotein	147/25%	**365**/**59%**	246/53%
α-Casein	743/33%	922/53%	**2176**/**54%**
β-Casein	1912/83%	5811/91%	**6785**/91%
Concanvalin A	2556/60%	4410/68%	**5660**/**73%**
α-Crystallin A	1792/60%	2748/80%	**2924**/**86%**
α-Crystallin B	687/66%	**1366**/**93%**	1203/79%
Cytochrome c	712/81%	**2176**/92%	1984/**94%**
GAPDH	525/33%	1815/63%	**2039**/**66%**
β-Lactoglobulin	527/30%	**1050**/**66%**	791/60%
Myoglobin	53/22%	813/51%	**1010**/**68%**
Ribonuclease A	41/19%	**488**/27%	476/**28%**
Thioredoxin	2962/67%	6023/**91%**	**6104**/88%
Transferrin	2167/26%	4691/46%	**5694**/**50%**

One pmol of starting material was analyzed by nanoUHPLC-MS using a 1 h LC gradient and an LTQ-Orbitrap XL mass spectrometer. Mascot ion scores (Score) and sequence coverages (SC) are displayed for the individual proteins searching the same data set with full N-terminal cleavage at serines and threonine (Full), semi-specific cleavage (Semi), and no enzyme (no). Highest score and sequence coverages are displayed in bold for the individual proteins

Bottom-up analysis is typically performed to confirm the protein sequence of therapeutic proteins. High sequence coverage of the protein is desirable to verify the identity of the sequence or modifications of it. However, high sequence coverage is dependent on the protein sequence and the available endoproteases. Recombinant granulocyte colony-stimulating factor (G-CSF) produced in *E. coli* (Filgrastim) is in clinical use since 1991 to reduce the duration and severity of neutropenia in patients undergoing myelosuppressive chemotherapy and to mobilize peripheral blood progenitor cells. Using trypsin, only a sequence coverage of 20% was obtained, because this protein with 174 amino acids contains a long region (42–147) without any arginine or lysine, respectively. Cleavage with Sc(III) triflate revealed a sequence coverage of 95% with semi-specific N-terminal serine/threonine cleavage (individual Mascot ion scores > 13).

### Cleavage of more complex protein samples

The immune-20S proteasome is a 700 kDa complex and contains 17 different protein subunits. Database search analysis of the immune-20S proteasome (from UBPBio) after exposure to Sc(III) triflate using Mascot against human Swiss-Prot database identified 13 proteins with ion scores above identity or extensive homology with full N-terminal serine/threonine specificity, 12 proteins with semi-N-terminal serine/threonine specificity, and 10 proteins with no enzymatic specificity (Table 2). Sixteen proteins of the immune-20S proteasome were previously identified after tryptic digestion using the same LC–MS system, but after separation of the proteins using 2D gel electrophoresis [21]. This result showed that Sc(III) triflate can perform similarly well as trypsin for the analysis of small protein complexes. Next, we analyzed 1 µg of the Universal Proteomics Standard (UPS1 from Sigma-Aldrich) which corresponds to 0.83 pmol each of 48 human proteins. Using trypsin, 44 proteins were identified, whereas 32 proteins with ion scores above identity or extensive homology with full N-terminal serine/threonine specificity, 27 proteins with semi-N-terminal serine/threonine specificity, and 25 proteins with no enzymatic specificity were found using Sc(III) triflate. This result revealed that Sc(III) triflate might be less suitable with increased complexity because of the limited cleavage specificity. We also tried to identify proteins after separation using SDS-PAGE, but have not been able to identify any protein. To explain this result, we combined an unstained gel piece and added one of the standard proteins (human transferrin) and incubated it with Sc(III) triflate and again could not identify any peptide. Therefore, it can be concluded that Sc(III) triflate is inactivated by the gel and is not applicable for in-gel digests.

**Table 2:** Immune-20S proteasome incubated with 100 mM Sc(III) triflate

Protein	Score/SC full (cut-off 22)	Score/SC semi (cut-off 33)	Score/SC no (Cut-off 41)
PSA1	150/7%	**178**/**11%**	147/**11%**
PSA2	154/**23%**	**166**/17%	107/13%
PSA3	**99**/10%	97/**14%**	64/10%
PSA4	63/4%	**209**/**14%**	123/11%
PSA5	177/**30%**	**274**/24%	188/24%
PSA6	**154**/**13%**	137/**13%**	112/**13%**
PSA7	76/**18%**	**93**/15%	
PSB1	**43**/**5%**	39/**5%**	
PSB2	**46**/**9%**	34/4%	97/7%
PSB3	130/**12%**	**166**/9%	145/9%
PSB4	95/**13%**	**140**/10%	117/10%
PSB8	**28**/**2%**		
PSB10	35/**8%**	**79**/6%	75/6%

1 µg of starting material was analyzed by nanoUHPLC–MS using a 1 h LC gradient and an LTQ-Orbitrap XL mass spectrometer. Mascot ion scores (score) are displayed for the individual proteins searching the same data set with full N-terminal cleavage at serines and threonine (Full), semi-specific cleavage (Semi), and no enzyme (no) against human Swiss-Prot database. Highest score and sequence coverages are displayed in bold for the individual proteins

## Conclusions

Sc(III) triflate is able to cleave proteins in-solution with predominant cleavage N-terminal to serine and threonine residues. The reagent worked with all proteins under investigation under more moderate temperatures and reduced reaction times than previously reported for the cleavage of peptides. Notably, the reagent is stable, relative cheap in comparison to endoproteinases, and the results were highly reproducible. Therefore, Sc(III) triflate can be a useful regent for the analysis of proteins, in particular for single protein analysis where classical endoproteinases as trypsin give limited sequence coverages because of the lack of cleavage sites.

## Electronic supplementary material

Below is the link to the electronic supplementary material.

Supplementary material 1 (PDF 556 kb)

## Abbreviations

G-CSF: Recombinant granulocyte colony-stimulating factor
LC–MS: Liquid chromatography–mass spectrometry
MALDI–MS: Matrix-assisted laser desorption/ionization mass spectrometry
MS: Mass spectrometry
Sc(III) triflate: Scandium(III) trifluoromethanesulfonate
UPS: Universal proteomics standard

## References

[1] Vandermarliere E, Mueller M, Martens L (2013). Mass Spectrom Rev.

[2] Giansanti P, Tsiatsiani L, Low TY, Heck AJ (2016). Nat Protoc.

[3] Trevisiol S, Ayoub D, Lesur A, Ancheva L, Gallien S, Domon B (2016). Proteomics.

[4] Swaney DL, Wenger CD, Coon JJ (2010). J Proteome Res.

[5] Smith BJ (1994). Methods Mol Biol.

[6] Villa S, De Fazio G, Canosi U (1989). Anal Biochem.

[7] Ni J, Kanai M (2016). Top Curr Chem.

[8] Miskevich F, Davis A, Leeprapaiwong P, Giganti V, Kostic NM, Angel LA (2011). J Inorg Biochem.

[9] Milovic NM, Dutca LM, Kostic NM (2003). Inorg Chem.

[10] Tanabe K, Taniguchi A, Matsumoto T, Oisaki K, Sohma Y, Kanai M (2014). Chem Sci.

[11] Elashal HE, Raj M (2016). Chem Commun (Camb).

[12] Seki Y, Tanabe K, Sasaki D, Sohma Y, Oisaki K, Kanai M (2014). Angew Chem Int Ed Engl.

[13] Lampi KJ, Ma Z, Hanson SR, Azuma M, Shih M, Shearer TR, Smith DL, Smith JB, David LL (1998). Exp Eye Res.

[14] Lyons B, Kwan AH, Truscott RJ (2016). Aging Cell.

[15] Kita Y, Nishii Y, Higuchi T, Mashima K (2012). Angew Chem Int Ed Engl.

[16] Ni J, Sohma Y, Kanai M (2017). Chem Commun (Camb).

[17] Koehler CJ, Strozynski M, Kozielski F, Treumann A, Thiede B (2009). J Proteome Res.

[18] Thiede B, Koehler CJ, Strozynski M, Treumann A, Stein R, Zimny-Arndt U, Schmid M, Jungblut PR (2013). Mol Cell Proteomics.

[19] Perez-Riverol Y, Csordas A, Bai J, Bernal-Llinares M, Hewapathirana S, Kundu DJ, Inuganti A, Griss J, Mayer G, Eisenacher M, Perez E, Uszkoreit J, Pfeuffer J, Sachsenberg T, Yilmaz S, Tiwary S, Cox J, Audain E, Walzer M, Jarnuczak AF, Ternent T, Brazma A, Vizcaino JA (2019). Nucleic Acids Res.

[20] Andreatta M, Alvarez B, Nielsen M (2017). Nucleic Acids Res.

[21] Schmidt F, Dahlmann B, Hustoft HK, Koehler CJ, Strozynski M, Kloss A, Zimny-Arndt U, Jungblut PR, Thiede B (2011). Amino Acids.

