# Supplementary material for: Predominant cleavage of proteins N-terminal to serines and threonines using scandium(III) triflate
Source: J Biol Inorg Chem. 2019 Oct 30;25(1):61–6. doi: 10.1007/s00775-019-01733-7 (PMC7064626; doi:10.1007/s00775-019-01733-7)

Predominant cleavage of proteins N-terminal to serines and threonines using scandium(III) triflate

Christian J. Koehler, and Bernd Thiede[#]

Supplementary figure 1: Peptide mass fingerprints of 12 single proteins using Sc(III) triflate.

The analysis was performed with MALDI-TOF-MS using 1 pmol of each protein. The most abundant corresponding peptide sequences obtained by LC-MS searching for semi-specific N-terminal cleavage at serine and threonine residues are shown including previous and following amino acid. Serine and threonine residues due to N-terminal cleavage are displayed with red letters as well as peptides from the protein C-terminal end (-). Overlapping peptide sequences within the spectra are shown with matching colored letters.

Alpha 1-acid glycoprotein

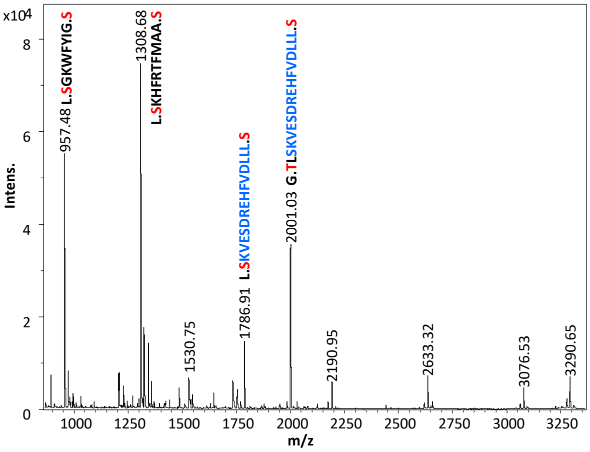

Concanvalin

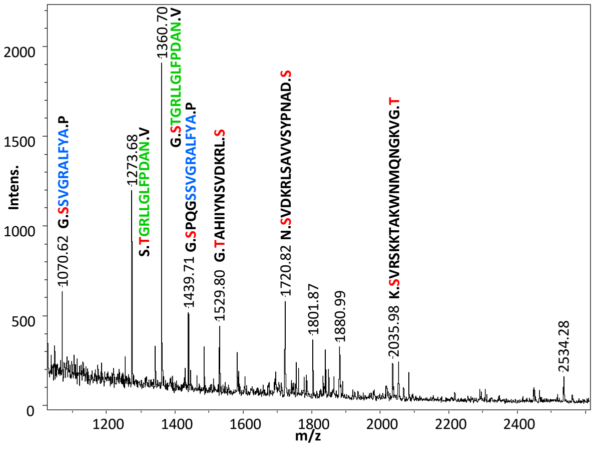

Alpha-casein

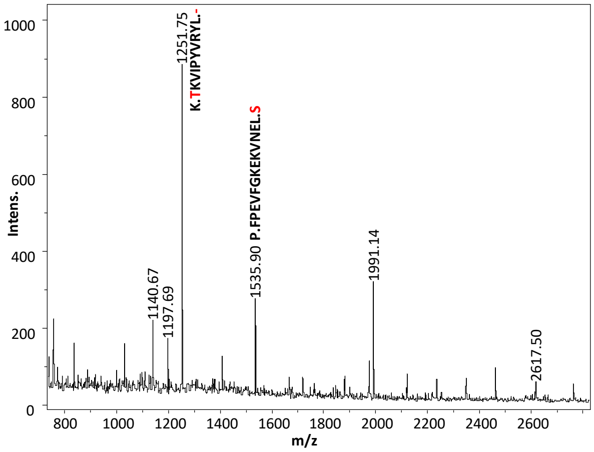

Alpha-crystallin

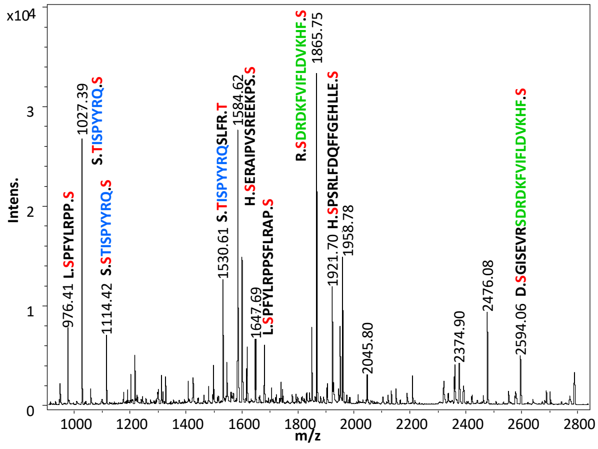

Beta-casein

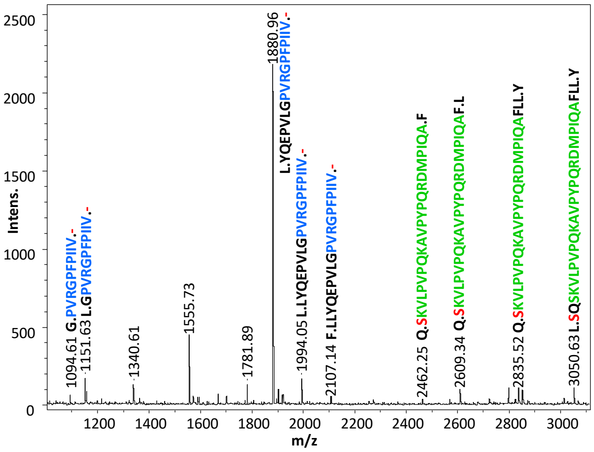

Cytochrome c

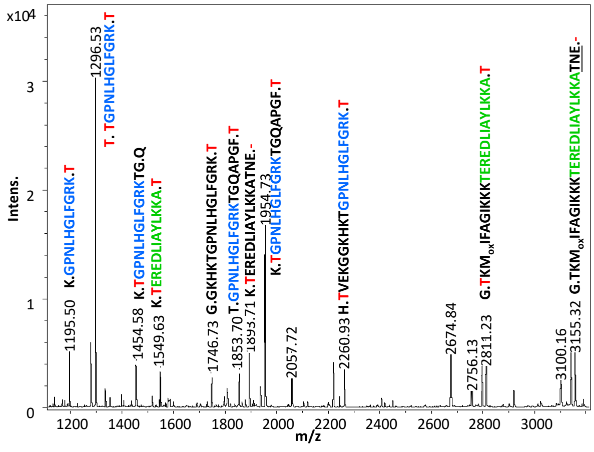

GAPDH

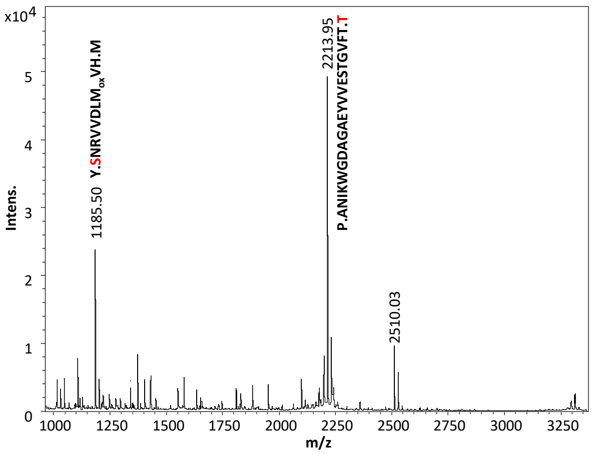

Ribonuclease A

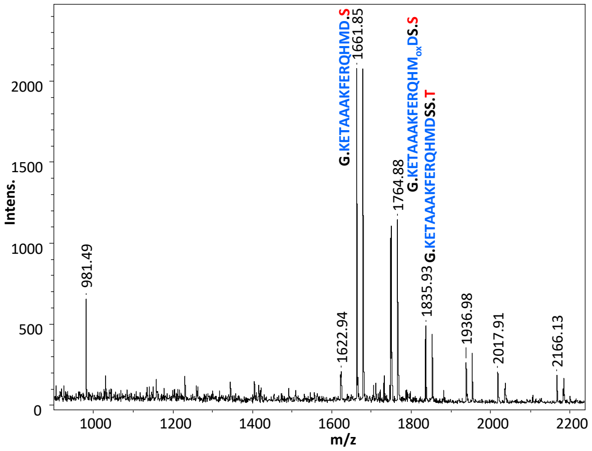

Beta-lactoglobulin

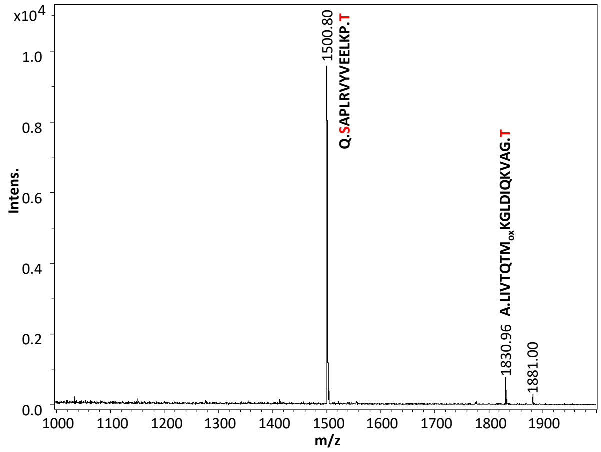

Thioredoxin

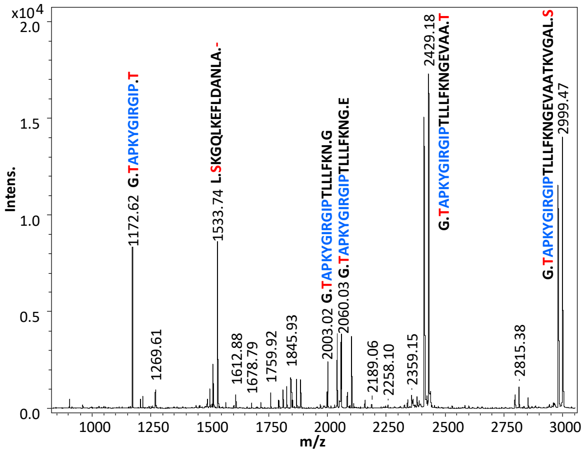

Myoglobin

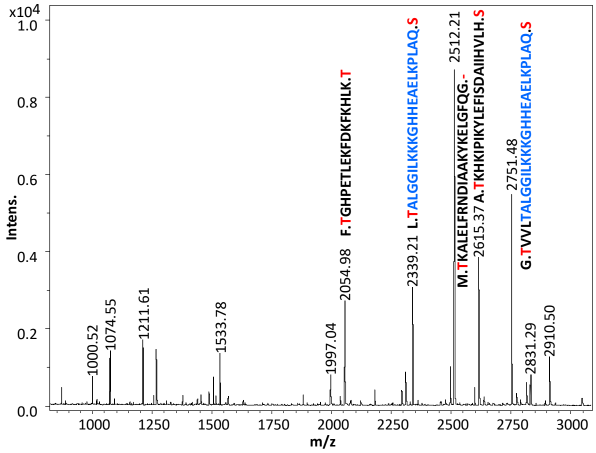

Transferrin

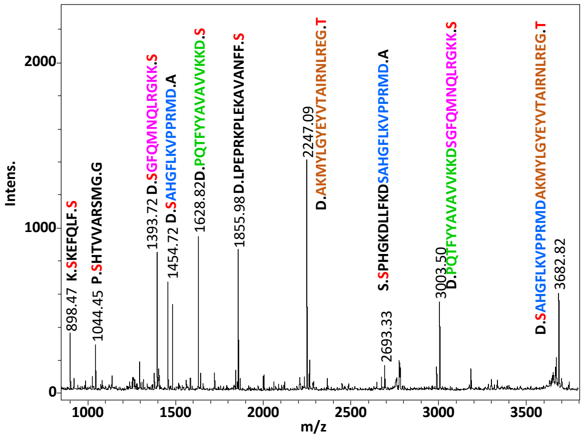

Supplementary figure 2: Sc(III) triflate cleavage sequence motif analysis.

GibbsCluster was applied to 2,470 unique identified peptide sequences including previous and following amino acid. To cover all peptides, motif length 10 (average peptide length), maximal insertion length 1 (smallest peptides contained nine amino acids) and maximal deletion length 39 (largest peptide contained 49 amino acids) was chosen for GibbsCluster analysis. N=2 corresponds to the N-terminal amino acid and N=9 to the C-terminal amino acid.

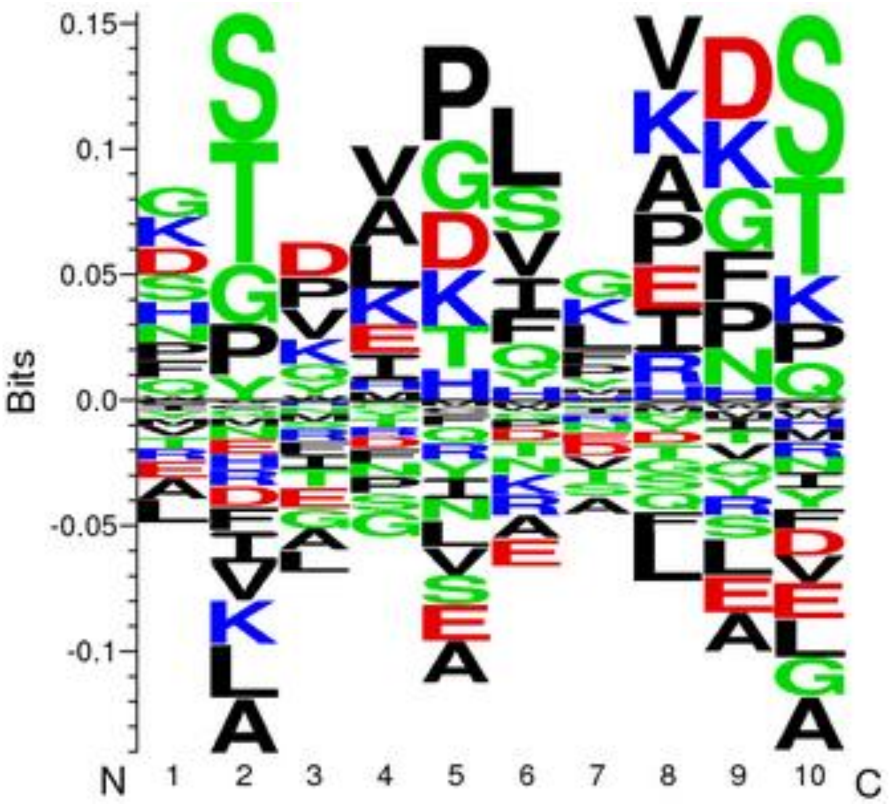

Supplement: Supplementary file 1 — Supplementary material 1 (PDF 556 kb) [file 775_2019_1733_MOESM1_ESM.pdf]
